# In reply to Cheng et al. (DOI: 10.1186/s13550-023-00965-8)

**DOI:** 10.1186/s13550-023-01018-w

**Published:** 2023-08-17

**Authors:** Efsun Somay, Erkan Topkan, Berrin Pehlivan, Ugur Selek

**Affiliations:** 1 Department of Oral and Maxillofacial Surgery, Faculty of Dentistry, Baskent University, 82. Street No: 26, Bahcelievler, Ankara Turkey; 2 Department of Radiation Oncology, Faculty of Medicine, Baskent University, Adana, Turkey; 3 Department of Radiation Oncology, Faculty of Medicine, Bahcesehir University, Istanbul, Turkey; 4 Department of Radiation Oncology, Faculty of Medicine, Koc University, Istanbul, Turkey

To the Editor

Cheng and colleagues' article entitled 'The added values of 18F-FDG PET/CT in differentiating cancer recurrence and osteoradionecrosis of mandible in patients with treated oral squamous cell carcinoma' was read with great interest and enthusiasm [1]. The present retrospective study was designed to investigate the potential clinical utility of 18F-FDG PET/CT functional parameters in discriminating between mandibular osteoradionecrosis (ORN) and cancer recurrence in 103 oral squamous cell carcinoma (OSCC) patients with suspected jaw ORN. Following histopathologic diagnosis, all subjects underwent PET/CT imaging with 18F-FDG within six months. Using receiver operating characteristic curve analysis and multivariable Cox regression models, clinical and imaging indicators of mandibular recurrence-free survival (MRFS) were determined after the acquisition of PET data. Recurrence of mandibular cancer was found in 24 (23.3%) individuals. The results of multivariate Cox regression analyses indicated that age at diagnosis of ≤ 52 years (*P* = 0.013), a location of the SUVmax voxel with soft tissue predominance (*P* = 0.019), and mandibular total lesion glycolysis (TLG) > 62.68 g (*P* < 0.001) were significant independent risk factors for MRFS. A risk scoring system for MRFS was developed, with scores ranging from 0 (no risk factor) to 3 (all three risk factors present). High-risk patients with a score of 2–3 had a substantially increased incidence of mandibular cancer recurrence than patients with a score of 0–1 (hazard ratio: 32.50, *P* < 0.001). Hence, the authors concluded that their scoring system possessed clinical utility in detecting mandibular cancer recurrence among patients exhibiting suspected ORN. The outcomes of the study are noteworthy and pioneering. However, we would like to express our apprehensions, which can potentially serve as a framework for forthcoming investigations when employed in tandem with the methodology employed by the researchers.

Prior research has established varying doses of radiotherapy (RT) as a critical factor in the risk of ORN, particularly concerning the maximum exposure of the jaw [2]. While some authors regard ≥ 60 Gy as the critical threshold [2, 3], others deem the V50 (volume exposed to 50 Gy or higher) of the mandible as the critical volumetric cutoff [3]. Although the mandibular mean, median, and Vx are likely the most reliable RT-related factors in determining ORN risk [4, 5], Cheng et al. [1] did not provide data on jaw dosimetry. The prescribed doses to the tumors were given as a dosimetric parameter, but depending on the RT technique and use of mandibular dose constraints, the mandible may receive significantly lower than the prescribed dose of ≥ 50 Gy [5]. Therefore, we believe that a more potent scoring system for distinguishing between ORN and tumor recurrences could be developed by integrating the robust dosimetric factors associated with RT, particularly those related to the jaw, as potential risk factors and categorizing patients accordingly. Subsequent research endeavors that concentrate on supplementary parameters could enhance our capacity to establish more dependable distinctions between tumor recurrences and severely incapacitating ORN, and potentially formulate prophylactic strategies against the ORN.

## Data Availability

Not applicable.

## Abbreviations

ORN: Osteoradionecrosis
OSCC: Oral squamous cell carcinoma
MRFS: Mandibular recurrence-free survival
TLG: Mandibular total lesion glycolysis
RT: Radiotherapy

## References

[1] Cheng NM, Lin CY, Liao CT, Tsan DL, Ng SH, Yen TC (2023). The added values of ^18^F-FDG PET/CT in differentiating cancer recurrence and osteoradionecrosis of mandible in patients with treated oral squamous cell carcinoma. EJNMMI Res.

[2] Nabil S, Samman N (2012). Risk factors for osteoradionecrosis after head and neck radiation: a systematic review. Oral Surg Oral Med Oral Pathol Oral Radiol.

[3] Tsai CJ, Hofstede TM, Sturgis EM, Garden AS, Lindberg ME, Wei Q (2013). Osteoradionecrosis and radiation dose to the mandible in patients with oropharyngeal cancer. Int J Radiat Oncol Biol Phys.

[4] Caparrotti F, Huang SH, Lu L, Bratman SV, Ringash J, Bayley A (2017). Osteoradionecrosis of the mandible in patients with oropharyngeal carcinoma treated with intensity-modulated radiotherapy. Cancer.

[5] Topkan E, Kucuk A, Somay E, Yilmaz B, Pehlivan B, Selek U (2023). Review of osteoradionecrosis of the jaw: radiotherapy modality, technique, and dose as risk factors. J Clin Med.

